# Managing non-acute subdural hematoma using liquid materials: a Chinese randomized trial of middle meningeal artery treatment (MAGIC-MT)—protocol

**DOI:** 10.1186/s13063-023-07608-2

**Published:** 2023-09-14

**Authors:** Qiao Zuo, Wei Ni, Pengfei Yang, Yuxiang Gu, Ying Yu, Heng Yang, Charles B. L. M. Majoie, Mayank Goyal, Jianmin Liu, Ying Mao, on behalf of MAGIC-MT investigators

**Affiliations:** 1 Neurovascular Center, Changhai Hospital, Naval Medical University, Shanghai, China; 2 Department of Neurosurgery, Huashan Hospital, Fudan University, Shanghai, China; 3 Department of Radiology & Nuclear Medicine, Amsterdam University Medical Center, Location University of Amsterdam, Amsterdam, The Netherlands; 4 Departments of Clinical Neuroscience and Radiology, Hotchkiss Brain Institute, Cummings School of Medicine, University of Calgary, Calgary, AB Canada

**Keywords:** Chronic subdural hematoma, Sub-acute subdural hematoma, Middle meningeal artery, Embolization, Recurrence, Progression

## Abstract

**Background:**

The conventional treatments for non-acute subdural hematoma (SDH) are facing the challenge of high hematoma recurrence and progression. A novel treatment of middle meningeal artery (MMA) embolization showed the potential role in decreasing the recurrence and progression rate of SDH compared to conventional treatments in multiple cohort studies. A randomized controlled trial is warranted to determine the effectiveness and safety of MMA embolization for non-acute hematoma and whether MMA embolization is superior to conventional treatments to lower the symptomatic recurrence and progression rate of non-acute SDH.

**Methods:**

This is an investigator-initiated, multi-center, prospective, open-label parallel group trial with blinded outcome assessment (PROBE design) assessing superiority of MMA embolization compared to conventional treatments. A total of 722 patients are planned to be randomized 1:1 to receive MMA embolization (intervention) or conventional treatments (control). The primary outcome is the symptomatic SDH recurrence/progression rate within 90 ± 14 days post-randomization.

**Discussion:**

This trial will clarify whether MMA embolization could reduce the recurrence or progression rate of symptomatic non-acute SDH compared to conventional treatment.

**Trial registration:**

ClinicalTrials.gov. Identifier: NCT04700345, Registered on 7 January 2021.

**Supplementary Information:**

The online version contains supplementary material available at 10.1186/s13063-023-07608-2.

## Administrative information

**Table:** 

Title {1}	Managing non-acute subdural hematoma using liquid materials: a Chinese randomized trial of middle meningeal artery treatment (MAGIC-MT) – Protocol
Trial registration {2a and 2b}	ClinicalTrials.gov Identifier: NCT04700345, Registered on 7 Jan 2021
Protocol version {3}	Date: 21 Sep 2022, Version 3.0
Funding {4}	The trial is funded by Shanghai Shenkang Hospital Development Center (SHDC2020CR1018B), Changhai Hospital (2020YSL004), Shanghai Municipal Health Commission (2023–62), and Covidien/Medtronic (20,212,016,387–01/1)
Author details {5a}	Qiao Zuo^1†^, Wei Ni^2†^, Pengfei Yang^1^, Yuxiang Gu^2^, Ying Yu^1^, Heng Yang^2^, Charles BLM Majoie^3^, Mayank Goyal^4^, Jianmin Liu^1^* and Ying Mao^2^*; on behalf of MAGIC-MT investigators^#^ † Qiao Zuo and Wei Ni are co-First authors * Correspondence: Ying Mao (maoying@fudan.edu.cn) and Jianmin Liu (liu118@vip.163.com) # The authors’ full names and affiliations are listed in Additional file 1
Name and contact information for the trial sponsor {5b}	Investigator initiated trial, principal investigators: Ying Mao (YM), Department of Neurosurgery, Huashan Hospital, Fudan University, 12 mid Wulumuqi Rd, Shanghai 200,040, China. Email: maoying@fudan.edu.cn Jianmin Liu (JL), Neurovascular Center, Changhai Hospital, Naval Medical University, 168 Changhai Rd, Shanghai 200,433, China. Email: liu118@vip.163.com
Role of sponsor {5c}	The trial sponsor, represented by YM and JL, is responsible for all aspects of conducting the trial including its design, data collection, management, analysis, interpretation of data, reporting results and the decision to submit the report for publication. Data safety is overseen by an independent Data Safety Monitoring Board (DSMB) consisting of a neurologist, a neuro-interventionist, and an independent methodologist/statistician not involved in the trial

## Introduction

### Background and rationale {6a}

Chronic subdural hematoma (cSDH) is one of the most common traumatic brain injuries, which is mainly found in the older population. It occurs in approximately 13.1–20.6 patients/100,000 and becomes more prevalent in patients aged > 65 years with an occurrence of 58.1 patients/100,000 [1, 2]. Its incidence is predicted to increase as a result of the aging population and the increased use of antiplatelet agents and anticoagulants by 2030 [3]. Surgical evacuation (burr-hole drainage or craniotomy) and medical treatment are the standard conventional treatments for cSDH. However, the high hematoma recurrence or progression rate of conventional surgical and conservative treatment cannot be ignored. The recurrence rate of hematoma after surgery ranges between 5.4 and 32% [4–8]. For conservative treatment, approximately 11.2% of atorvastatin-treated patients and 10% of dexamethasone-treated patients underwent surgery as a result of hematoma progression over the medication course [9, 10].

The potential mechanisms of hematoma recurrence or progression remain unclear. One of them is due to the underlying pathophysiologic mechanism of cSDH formation, which involves the formation of fragile capillaries along the subdural membrane encapsulating the collection [11, 12]. Thus, endovascular embolization of the middle meningeal artery (MMA) has emerged as a promising treatment modality to lower the hematoma growth and recurrence rates by eliminating the vascular supply to the membrane of cSDH [13]. A number of observational studies evaluating the safety and efficacy of MMA embolization as a stand-alone procedure or in combination with surgical evacuation have shown favorable outcomes in comparison to conventional therapies. Several preliminary cohort studies reported that the rate of incomplete hematoma resolution or surgical rescue in MMA embolization group (ranging 1.4 to 8.9%) was significantly lower than that in the conventional treatment group (ranging 14% to 27.5%) without difference of procedure-related complication rate between the two modalities [14–18]. A systematic review reported that the recurrence rate of patients treated with MMA embolization for recurrent cSDH was 2.4% and the recurrence rate for primary cSDH was 4.1% [19]. Another meta-analysis reported a markedly lower recurrence rate (2.1% vs. 27.7%) and similar complication rate for cSDH after MMA embolization compared with conventional management, and the recurrence rate of six MMA embolization single-arm series was 3.6% [20]. We reported the first Chinese cSDH case series of 6 cases with MMA embolization alone and 15 cases with MMA embolization combined with burr-hole drainage and found no hematoma recurrence or progression or procedure-related complications [21]. These preliminary results revealed a promising role of MMA embolization for both the single management and as a surgical adjuvant for many patients with cSDH, necessitating large-scale trials to examine the efficacy and safety of MMA embolization for cSDH in comparison to traditional treatments.

So far, there have been several multi-center randomized controlled trials (RCT) recruiting chronic and subacute SDH patients treated by MMA embolization in Europe and North America. The randomized trial of MMA treatment (MAGIC-MT) aims to assess whether additional MMA embolization using liquid materials can produce lower hematoma progress/recurrence rate than conventional treatment for non-acute (chronic and subacute) SDH in a Chinese population.

### Objectives {7}

This study aims to assess whether additional MMA embolization using liquid materials can produce lower hematoma progress/recurrence rate than conventional treatment for non-acute (chronic and subacute) SDH in a Chinese population.

### Trial design {8}

**Fig. 1:**
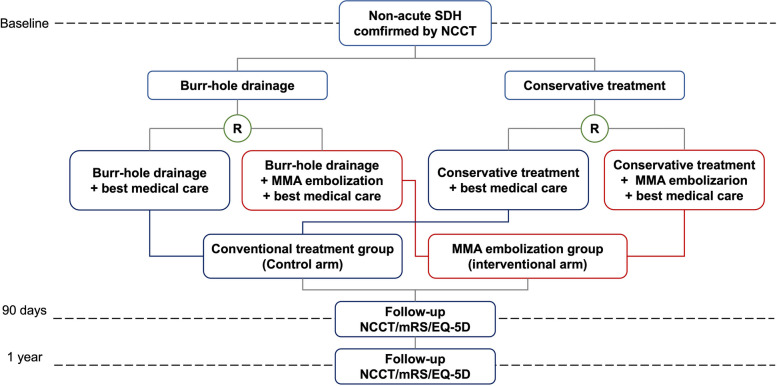
Patient flow

MAGIC-MT is a multicenter phase III prospective randomized clinical trial with open-label treatment and blinded outcome assessment (PROBE) to assess the superiority of MMA embolization compared to conventional treatments. The intervention is MMA embolization, and the comparator is conventional treatment with best medical care. Patients are randomized 1:1. Patient flow is depicted in Fig. 1.

## Methods: participants, interventions, and outcomes

### Study setting {9}

The sponsors are Huashan Hospital affiliated to Fudan University and Changhai Hospital affiliated to the Naval Medical University, China. As a quality initiative, all the centers should meet the following minimum criteria: (1) the center should be the local tertiary hospital, (2) the center should have experience in conducting brain trauma or neurovascular disease trials, and (3) the center could carry out both burr-hole drainage and MMA embolization and have more than 50 cases of cSDH every year. Currently, 31 centers (Additional file 1) in China are recruiting patients for the study. A document detailing the organizational structure behind the MAGIC-MT trial, including all committees and a list of the MAGIC-MT investigators is added to Additional file 1.

### Eligibility criteria {10}

#### Inclusion criteria

Patients can be included in MAGIC-MT if they are over 18 years of age and have a symptomatic non-acute SDH with mass effect and independent functional status with mRS score ≤ 2 prior to symptom onset.

#### Exclusion criteria

The exclusion criteria are as follows: (1) with massive cerebral infarction, intracranial tumor, or mass lesion; (2) need craniotomy or emergent SDH evacuation; (3) bilateral SDH with unknown origin of symptoms; (4) recurrent SDH; (5) any sign of anatomical variations that could make MMA embolization unsafe (e.g., prominent MMA-ophthalmic artery anastomoses); (6) INR > 1.5 and/or platelet count < 80 x10^9/L; (7) contraindications for angiography; (8) pregnancy; and (9) life expectancy < 1 year.

### Who will take informed consent? {26a}

Investigators should adequately explain the details of the clinical trial, including known, foreseeable risks and possible adverse events, etc., to the subjects or to the guardians of subjects without capacity for civil conduct. After full and detailed explanation, subjects or their guardians, as well as the investigators, sign their name and date on the informed consent form. As part of the cSDH patients are expected to suffer from aphasia, lack understanding of their disease, or have other acute cognitive symptoms, we have determined that if (1) the ethics committee agrees in principle and (2) investigators believe that participating in the clinical trial is in the subjects’ own interest, patients can enter the clinical trial if their guardians should sign the name and date before inclusion in the trial.

### Additional consent provisions for collection and use of participant data and biological specimens {26b}

N/A. Biological specimens collection are not needed in this trial.

## Interventions

### Explanation for the choice of comparators {6b}

Patients assigned to the control group will be managed according to the current standard of care, i.e., burr-hole drainage and/or best medical management. The standard burr-hole drainage is performed under general or monitored local anesthesia. The hematoma is evacuated with plenty irrigation after which a subdural drain is placed into the subdural space. The drain is removed within several days according to the CT scan and drainage volume which is decided by the treating physician. For best medical care, it is recommended to administer drugs (e.g., atorvastatin or dexamethasone) for patients without drug contraindications [9, 22, 23]. The data of conventional treatment measures are recorded.

### Intervention description {11a}

Patients randomized to MMA embolization group will receive cerebrovascular angiography and ipsilateral MMA embolization of the SDH. Patients are under either general anesthesia or monitored local anesthesia. Intravenous heparin administration during the procedure will be recorded. Briefly, femoral or radial artery access is obtained, and digital subtraction angiography (DSA) for cerebrovascular assessment is performed using a standard 5 French diagnostic catheter. Then, a guiding catheter is placed in the proximal external carotid artery. A microcatheter is advanced selectively under roadmap guidance into the main trunk of MMA, and MMA superselective angiography is performed to evaluate for potentially dangerous collateral vessels such as ophthalmic and petrous branches prior to embolization [13]. After identifying no collaterals, the MMA is embolized with ONYX-18 (Medtronic, USA) under a blank fluoroscopic roadmap. The successful embolization is defined as that both frontal and parietal branches are selectively embolized or the main trunk of MMA was embolized. For complete embolization, it is recommended that the microcatheter is navigated distally in the frontal and parietal branch to ensure the embolic materials penetrating into the small vessels as much as possible. MMA embolization should be conducted before the burr-hole drainage if patients are allocated to the MMA embolization combined with burr-hole drainage group in this trial.

### Criteria for discontinuing or modifying allocated interventions {11b}

Patients may withdraw their consent at any time without providing a reason and thus terminate their participation in the study prematurely. Withdrawal from the study and reasons, if known, will be documented. Criteria for premature drop-out include the following: subsequent occurrence of an exclusion criterion, loss of contact, death, and declaration of withdrawn consent.

### Strategies to improve adherence to interventions {11c}

N/A. The intervention of MMA embolization is a very common technique and has been long used for intracranial arteriovenous malformation or fistula, so we think that there is no possibility to influence patients’ adherence to the intervention.

### Relevant concomitant care permitted or prohibited during the trial {11d}

N/A. Patients in both groups are treated according to the currently standard of care, and any concomitant care as part of routine clinical practice is permitted.

### Provisions for post-trial care {30}

A proband cover for all participants in the trial is contracted to compensate for trial-associated harm occurring within the final study visit.

### Outcomes {12}

#### Primary outcome

The primary outcome is the symptomatic SDH recurrence/progression rate within 90 ± 14 days post-randomization. “Symptomatic” is defined as one or more of the following features which are attributed to the progression/recurrence: headache, short-term cognitive decline, speech difficulty or aphasia, gait disturbance, focal weakness, sensory deficits, and seizures. SDH recurrence is defined as the maximum thickness of SDH exceeding 10 mm or the patient receiving re-operation in patients who underwent burr-hole drainage. SDH progression is defined as the maximum thickness of SDH increasing over 3 mm compared to the baseline or the patient receiving surgical rescue in patients who received conservative treatment.

#### Secondary outcomes

The secondary outcomes are as follows: (1) SDH recurrence/progression rate at 1 year; (2) successful embolization rate of the target vessels based on DSA; (3) change in hematoma thickness at 90 days compared to baseline; (4) change in hematoma volume at 90 days compared to baseline; (5) change in midline shift at 90 days compared to baseline; (6) change in the mRS at 90 days and 1 year compared to baseline; (7) mRS of 0 to 3 at 90 days and 1 year; (8) mRS of 0 to 2 at 90 days and 1 year; (9) quality of life assessed by EQ-5D scale 0 (worst health) to 100 (best health) at 90 days and 1 year [24].

### Participant timeline {13}

All trial procedures and radiological and clinical visits are summarized in Table 1.

**Table 1:** Timing of all procedures in MAGIC-MT

Timepoint	-*t*_1_	0	30 days	90 days	1 year
Enrollment					
Eligibility screen	X				
Informed consent	X				
Allocation		X			
Interventions					
MMA embolization		X			
Conventional treatment		X			
Assessment					
Medical history	X				
Vital signs	X		X	X	X
Baseline NCCT	X				
DSA imaging		X			
Procedure details		X			
NCCT follow-up				X	X
mRS	X			X	X
EQ-5D	X			X	X

### Sample size {14}

Our estimates are based on the hematoma recurrence or progression rate of conventional treatment from the literature. The hematoma recurrence or progression rate of conventional treatment ranged from 14 to 27.5% [14–18], and the hematoma progression rate of atorvastatin treatment is 11.2% as reported [9], so we assume the event rate of the control group is 12%. The hematoma recurrence of MMA embolization ranged from 1.4 to 8.9% as reported [14–18], and a systematic review reported that the recurrence rate for patients treated with MMA embolization for primary cSDH was 4.1% [19], so we assume the event rate of the intervention group is 5%. The trial is powered to assess superiority. When assuming the event rate of 5% in intervention group and 12% event rate in the control group, with a power of 90% and two-sided alpha of 0.05, allowing for 8% drop-outs, the estimated sample size is 722 patients in total.

### Recruitment {15}

The clinical investigators will be trained in communicating with potential participants and their relatives, documentation including screening logs, and other standard operating procedures during the kick-off meeting of each center. All the centers will recruit patients competitively, and recruitment progress will be reported weekly to the steering committee to track the recruitment process. To ensure the sufficient enrolled patients, all the centers will expand the partnership with local hospitals and encourage the referrals of potential subjects. The estimated rate of recruitment is 3 to 5 patients per month in centers in big cities and 1–2 patients in medium-sized city centers, and the expected recruitment time will last 2 years.

## Assignment of interventions: allocation

### Sequence generation {16a}

After a non-acute SDH has been confirmed and written informed consent form has been obtained, randomization is allowed. The randomization procedure is computer- and web-based and stratified by burr-hole drainage surgery.

### Concealment mechanism {16b}

Participants are randomized using Bioknow (Bioknow eClinical Solutions, Beijing, China), a web-based, GCP-compliant electronic data capture (EDC) system for collecting patient data in clinical trial, observational studies, and registries. It maintains allocation concealment as it does not release the randomization code until screening has been completed and the patient was cleared to be recruited onto the trial.

### Implementation {16c}

Extended stratified block algorithms generate an unpredictable allocation sequence. Random assignment by Bioknow cannot be influenced by clinical investigators.

## Assignment of interventions: blinding

### Who will be blinded {17a}

As this is an open-label trial, the treatment allocation is known to both the treating physician and the patient. A blinded, trained investigator in each center will procure the information concerning clinical outcome at 3 months using standardized forms and procedures. Based on these reports, members of the outcome committee who are blinded for treatment allocation assign the final score on the modified Rankin and EQ-5D scale. Neuroimaging will be also assessed by a core-laboratory blinded for treatment allocation. To report to the data safety monitoring board (DSMB), outcome data will be combined with data on treatment allocation by an independent trial statistician. Members of the steering committee are kept blinded of results of interim analyses of efficacy and safety.

### Procedure for unblinding if needed {17b}

N/A. The design is open label with only outcome assessors being blinded, so unblinding will not occur.

## Data collection and management

### Plans for assessment and collection of outcomes {18a}

Data will be entered by clinical investigators and supporting trial personnel on electronic case report forms (eCRFs). The size and extent of cSDH will be measured on non-contrast CT scans. The modified Rankin scale has an additional grade ranging from 0 (no symptoms) to 6 (dead) and is used to value the neurologic function. The EQ-5D questionnaire is used to describe and value the health status.

### Plans to promote participant retention and complete follow-up {18b}

Investigators, doctors, and nurses will take very care of the participants in the trial until the scheduled last follow-up visits. The transportation expenses for the follow-up are reimbursable. Besides, patients will also be explained there is scientific evidence that patients treated in a clinical trial environment may show superior outcomes compared to those managed under routine practice conditions.

### Data management {19}

Final data capture and storage will be done via eCRFs using an electronic database. Investigators and trial staff will be introduced to the platform and trained in data entry during the initial kick-off meeting prior to recruitment of the first patient. Only authorized clinical investigators and trial personnel will be granted access to the study database by a personal ID. Depending on their role within the study, users will be assigned tailored authorizations to the respective forms.

### Confidentiality {27}

When adding a new patient to the database, identifying data are entered on a form which is only printed but not saved on the server. On this form, the participant’s name consists of a combination of four English alphabet (the initial letter of Chinese name pronunciation). The form is kept in a locked space to which only the principal investigator has access and may be used to unblind personal data if necessary.

### Plans for collection, laboratory evaluation, and storage of biological specimens for genetic or molecular analysis in this trial/future use {33}

N/A. See the “ Additional consent provisions for collection and use of participant data and biological specimens {26b}” section, and there will be no biological specimens collected.

## Statistical methods

### Statistical methods for primary and secondary outcomes {20a}

The primary analysis is to evaluate the symptomatic SDH recurrence/progression rate within 90 days post-randomization and to determine whether MMA intervention is superior to conventional treatment. It will be performed in the full analysis set following intention-to-treat principle, which will include all patients randomized in the study. A CMH chi-square test will be used as primary analysis to test the difference in primary outcome between the experimental and control groups, using treatment strata (burr-hole drainage/best medical management) as the stratification factor. Besides, odds ratio (OR) with 95% confidence intervals will be calculated by a logistic regression model. Binary second efficacy outcomes will be analyzed using the same method as primary endpoint analysis. For continuous outcomes, a linear mixed model with study center as random effect will be used for the analysis of hematoma thickness and hematoma volume. Other continuous outcomes will be analyzed by a two-sample *t* test.

For both primary and secondary outcomes, we also performed pre-specified subgroup analyses according to burr-hole drainage and best medical management. Safety analyses will be based on all randomized patients who have received study treatment. The numbers and percentages of subjects with any AEs or any SAEs will be summarized by treatment group. Subject death will be summarized and listed. All analyses will be performed by SAS system version 9.4.

### Interim analyses {21b}

The safety analysis will be performed when randomization is completed of the first 100 and 620 participants with a hospital admission of at least 1 week. The safety and efficacy interim analyses will be performed when randomization and 90-days follow-up are completed of 240 and 480 participants, respectively. Based on these analyses, the DSMB advises the steering committee if the randomized comparisons in MAGIC-MT have provided both (1) “proof beyond reasonable doubt” that for all, or specific subgroups of patients, one particular treatment is clearly beneficial or detrimental concerning a net difference in outcome, and (2) evidence reasonably expected to influence patient management. No precise criteria of proof beyond reasonable doubt can be formulated, but a difference between treatment arms of three standard deviations or more concerning outcomes might be needed to justify premature termination or modification of the trial.

### Methods for additional analyses (e.g., subgroup analyses) {20b}

Primary and secondary outcomes will be stratified for surgical treatment (burr-hole drainage or not) in a categorial manner.

### Methods in analysis to handle protocol non-adherence and any statistical methods to handle missing data {20c}

The primary analysis will be performed on the intent-to-treat set including all randomized patients and based on the treatment arm they were randomized to, regardless of the therapy they actually received. A missing data analysis will be conducted investigating the extent and type of missing values in trial endpoint variables. In case of a suspected non-random missing data mechanism, corresponding sensitivity analyses will be carried out along with a discussion of the results. If suitable, we will consider multiple imputation to fill empty cells in the dataset.

### Plans to give access to the full protocol, participant-level data, and statistical code {31c}

The trial was registered prospectively in ClinicalTrials.gov on 7 January 2021 with the Identifier NCT04700345. Updates will be submitted if significant milestones have been reached. The datasets analyzed during the current study and statistical code are available from the corresponding author on reasonable request, as is the full protocol.

## Oversight and monitoring

### Composition of the coordinating center and trial steering committee {5d}

The steering committee consists of four Chinese experts and two international experts outside China, and the two study coordinators will ensure high process quality and compliance with the study protocol. The independent clinical event committee will achieve high-quality outcomes and reduce inconsistencies or bias in clinical trial data. Participating clinicians must take part in a training course as part of a kick-off event initiated by the principal investigator. Each participant must confirm in written form that she/he was properly introduced to trial-specific procedures.

### Composition of the data monitoring committee, its role and reporting structure {21a}

The DSMB will regularly receive blinded statistical reports and monitor serious adverse events (SAEs) throughout the trial and decide whether patient safety is compromised, demanding premature closure of the trial. An initial meeting of the DSMB will be held in order for the DSMB members to fully understand the research protocol, to review and approve the DSMB charter, to review the monitor plans for safety and efficacy data, and to discuss the statistical methods, including the stopping rule. The DSMB plans to perform the safety analysis when randomization is completed of the first 100 and then 620 participants with a hospital admission of at least 1 week and two interim analysis meetings when randomization and 90-days follow-up are completed of 240 and 480 participants respectively. During patient enrollment, interim analyses of mortality and available major outcomes and other requested analyses will be supplied confidentially to the chairman of the DSMB. Besides the planned meetings, the DSMB will perform ongoing safety surveillance during the inclusion period of the first 50 participants, after which they will advise on how intensively to monitor the safety data. Any mortality or other serious adverse events will be directly reported to the DSMB and will be evaluated for possible relatedness to the study intervention. An ad hoc meeting of the DSMB may be called at any time by the principal investigators or the DSMB if imminent participants’ safety issues arise.

### Adverse event reporting and harms {22}

Adverse events (AEs) and serious adverse events (SAEs) are defined according to the ICH GCP guidelines [25]. All AEs and SAEs reported by study participants or observed by an investigator within the study period must be documented in the eCRF and be reported to DSMB, including but not limited to: death from any cause, any neurological deficit, any hemorrhagic complication, ischemic stroke and surgical infection as reported by the local principal investigator, allergic contrast reactions, and any hospital-acquired infections that lead to prolongation of existing inpatients’ hospitalization.

### Frequency and plans for auditing trial conduct {23}

The data management staff will stay in regular contact with the investigators about trial progress, data consistency, missing data, and time window violations. If necessary, data queries for missing data as well as clarifications of inconsistencies or discrepancies will be sent.

### Plans for communicating important protocol amendments to relevant parties (e.g., trial participants, ethical committees) {25}

Any change in the protocol must be cleared in written form and signed by all persons in charge, stating the reasons for changes. Subsequently, these changes will be considered part of the study protocol. If necessary, changes must also be approved by the Institutional Review Board. Changes must be filed as an amendment in the trial master file (TMF).

### Dissemination plans {31a}

After database closure, a biometric report will be written by the trial statistician describing the main study results. Subsequently, a meeting among investigators and collaborators will be held to discuss findings prior to drafting of a scientific manuscript to be submitted for peer-review and publication in a major scientific journal. Also, we will attempt to present results at key international conferences of neurosurgical and neurointerventional societies.

## Discussion

MAGIC-MT is a phase 3 randomized clinical trial with a PROBE design assessing superiority of MMA embolization compared to conventional treatment in the Chinese population concerning non-acute SDH recurrence and progression at 3 months.

Multiple RCTs are currently randomizing patients to receive MMA embolization or conventional treatment. The EMBOLISE trial (NCT04402632) with estimated enrollment of 600 patients in the USA has similar design to our protocol, using ONXY for the MMA embolization with primary outcome of incidence of hematoma recurrence/progression requiring re-intervention within 90 days post-procedure, which may allow for individual patient pooling of trial data after conclusion of the two trials in the future. This will result in increased power to detect small treatment effects and to explore effects in important subgroups. Besides, the STEM trial (NCT04410146), MEMBRANE trial (NCT04816591), and OTEMACS trial (NCT04742920) with MMA embolization using liquid materials are all recruiting participants in North America and Europe, and none of these trials is set-up to provide results generalizable to the Chinese population. In China, there is a domestic RCT trial (ChiCTR2000039359) titled “Endovascular therapy combined with operation and simple operation in the treatment of chronic subdural hematoma: a randomized controlled trial” registered on the Chinese Clinical Trial Registry Website, assessing the recurrence rate of MMA embolization plus burr-hole drainage compared to burr-hole drainage alone with the estimated sample size of 480 participants. Unlike this trial, our trial enrolls the symptomatic non-acute SDH regardless of the necessity for burr-hole drainage, with a more comprehensive representation of current conventional treatments in the control group, and the larger sample size also shows the advantages of our trial.

## Trial status

This manuscript is based on trial protocol version 3.0 dated 21 September 2022. The first patient was included in March 2021, and the last patient was enrolled in May 2023. According to the original plan, we thought we were able to finish the recruitment by April 2022. But due to the COVID-19 pandemic, many districts and cities in China have been affected and locked down in the past 2 years, which has seriously delayed the implementation and recruitment of this trial.

### Supplementary Information

**Additional file 1. **Centers in China recruiting patients for the study.

## Data Availability

Data will be made available from the corresponding author upon reasonable request.

## Abbreviations

cSDH: Chronic subdural hematoma
SDH: Subdural hematoma
MMA: Middle meningeal artery
RCT: Randomized controlled trial
DSMB: Data safety monitoring board
DSA: Digital subtraction angiography
NCCT: Noncontrast CT
mRS: Modified Rankin Scale
AEs: Adverse events
SAEs: Serious adverse events
MAGIC-MT: Managing non-acute subdural hematoma using liquid materials: a Chinese randomized trial of MMA treatment
TMF: Trial master file
eCRFs: Electronic case report forms
