# Supplementary material for: Managing non-acute subdural hematoma using liquid materials: a Chinese randomized trial of middle meningeal artery treatment (MAGIC-MT)—protocol
Source: Trials. 2023 Sep 14;24:586. doi: 10.1186/s13063-023-07608-2 (PMC10503047; doi:10.1186/s13063-023-07608-2)
Supplement: Supplementary file 1 — Additional file 1. Centers in China recruiting patients for the study. [file 13063_2023_7608_MOESM1_ESM.docx]

**Appendix I**

Ying Mao, Department of Neurosurgery, Huashan Hospital , Fudan University; Jianmin Liu, Neurovascular Center, Changhai Hospital, Naval Medical University; Yuxiang Gu, Department of Neurosurgery, Huashan Hospital, Fudan University (North Hospital); Jieqing Wan, Department of Neurosurgery, Renji Hospital Affiliated to Shanghai Jiaotong University School of Medicine; Liang Gao, Department of Neurosurgery, Shanghai Tenth People's Hospital; Xuming Hua, Department of Neurosurgery, Xinhua Hospital Affiliated to Shanghai Jiaotong University School of Medicine; Hengli Tian, Department of Neurosurgery, Shanghai Sixth People's Hospital; Yi Li, Department of Neurosurgery, Shanghai Ninth People's Hospital; Jing Luo, Department of Neurosurgery, The First Affiliated Hospital of Anhui Medical University; Zhenbao Li, Department of Neurosurgery, The First Affiliated Hospital of Wannan Medical College (Yijishan Hospital of Wannan Medical College); Weiwei Wang, Department of Neurosurgery, Zhangzhou Municipal Hospital of Fujian Province; Yuanxiang Lin, Department of Neurosurgery, The First Affiliated Hospital of Fujian Medical University; E Chen, Department of Neurosurgery, Zhongshan Hospital Xiamen University; Mingfa Liu, Department of Neurosurgery, Shantou Central Hospital; Jianming Wu, Department of Neurosurgery, Shenzhen Second People's Hospital; Conghui Li, Department of Neurosurgery, The First Hospital of Hebei Medical University; Tianxiao Li, Department of Neurosurgery, Henan Provincial People's Hospital; Wenyuan Zhao, Department of Neurosurgery, Zhongnan Hospital of Wuhan University; Jian Chen, Department of Neurosurgery, Affiliated Hospital of Nantong University; Ya Peng, Department of Neurosurgery, The First People's Hospital of Changzhou; Yong Zhen, Department of Neurosurgery, Northern Jiangsu People's Hospital; Qingrong Zhang, Department of Neurosurgery, Nanjing Drum Tower Hospital; Hua Lu, Department of Neurosurgery, Jiangsu Provincial Hospital; Meihua Li, Department of Neurosurgery, The First Affiliated Hospital of Nanchang University; Guohua Mao, Department of Neurosurgery, The Second Affiliated Hospital of Nanchang University; Jiyue Wang, Department of Neurosurgery, Brain Hospital of Liaocheng People's Hospital; Jianjun Yu, Department of Neurosurgery, Linyi People's Hospital; Jianmin Zhang, Department of Neurosurgery, The Second Affiliated Hospital Zhejiang University School of Medicine; Ming Zhong, Department of Neurosurgery, The First Affiliated Hospital of Wenzhou Medical University; Zhiqing Lin, Department of Neurosurgery, Ningbo First Hospital; Yang Wang, Department of Neurosurgery, Beijing Chaoyang Hospital, Capital Medical University.
